# Editorial Notes and Comments

**Published:** 1911-10

**Authors:** 


### Editorial Notes and Comments.

Dr. Vard H. Hulen has removed from Houston, Texas, to San Francisco, Cal.

The book reviews in this issue make good reading; they are all "editorial." Read 'em.

The Mississippi Valley Medical Association will meet in Nashville, October 17, 18, 19 (inst.).

Dr. J. S. Steele, of Monterey, Mexico, has gone to Vienna to take a special course on the eye, his specialty for many years. On his return he will remove to San Antonio.

Attractive.—See J. R. Reed Music Co.'s ad. Bully! They will sell you a darling Victriola, like mine, for $15; one dollar per week, if you wish. The perfection of the gramaphone—without horns!

Dr. J. G. Baldwin, of Honey Grove, Texas, has removed to San Antonio. He has gone to New York to take post-graduate instruction in diseases of the rectum and stomach, and will make such diseases his specialty.

Drs. Bennett and Scott, of Austin, announce that they will limit their practice in future to surgery and diseases of women. They have been remarkably successful with transfusion in pellagra. (See report of cases in *Texas State Journal of Medicine*, August.)

According to State Superintendent, Texas has made more progress in the past two years than any other State in the Union in the erection and equipment of schoolhouses. During the scholastic year ending August 31, 1910, 643 public schoolhouses were erected in common, and 127 in independent school districts. This is at the rate of over two houses per day at an average cost of $3340. During the same period $2,026,230 was expended for equipment and $691,889 for additions.

All Honor to Mr. Taft—for once: He has sustained Dr. Wiley and declines to ask for his resignation, upon the advice of Mr. Attorney General Wickersham. He sat down on the corporation lawyer,—the representative of those "interests" which, for seventeen years, defeated the passage of the Pure Food Bill, and which are now hounding Dr. Wiley. The accusation against Dr. Wiley that he paid an expert chemist a few dollars "more than the law stipulates" is puerile. Taft said, as did Pilate: "I find no guilt in this man"; yet the "interests"—like the howling mob—would still crucify him.

---

Pellagra: Another Guess.—Dr. G. C. Mizule, Ph. D., Atlanta, Ga., writing in the *Journal Record of Medicine,* June, 1911, says: "Pellagra is a disease due to eating semi-drying oils (as cottonseed oil, etc.) in quantities that can not normally be disposed of, in which case they are deposited as fat foreign to the human organism and in their final disposition (by oxidation) there is developed a series of products that act in a deleterious manner upon the cells and intracellular tissues of the body." And he says, furthermore, that sulphide of calcium in one-sixth-grain doses will cure it.

---

The Limit.—Prof. R. A. Millikan, of Chicago, has isolated and measured an ion,—the ultimate unit of an electrical discharge—the smallest thing in the world. It is so small that if one hundred millions of people should begin to collect and count these atoms [ions] of electricity * * * at the rate of 100 every minute * * * it would require about four millions of years to collect enough to generate by electrolysis sufficient hydrogen to fill a child's toy balloon eight inches in diameter. So says Wm. J. Humphreys, U. S. Weather Bureau, in *Scientific American,* September 2, 1911.

---

Euthanasia for the Leper.—The *Medical Sentinel*, commenting on Dr. R. H. L. Bibb's article on Leprosy in the July issue of the Texas Medical Journal, says:

"He [Dr. Bibb] states that the consensus of opinion among all prominent authorities is that the disease is absolutely incurable in any stage. Dr. Bibb makes this statement very emphatically: *'There is no living man that knows of any method by which a single case of leprosy can be cured.'* The only way of combating the scourge is by separation and segregation,—separation from the rest of humanity, and segregation in leper colonies. He advocates the separate segregation of the sexes. The interesting article of Dr. O'Day's in the August *Sentinel* seems also so imply that such a course of action would be advisable to prevent leprous marriages.

"Leprosy is a disease with which we of the Northwest are to a large extent happily unfamiliar. Its loathsomeness, its hideous progress, slow as the anger of the gods, but as certain as fate, these are matters with which most of us have only a literary acquaintance. But if the disease is positively incurable, if every leper is completely without hope, and is, besides that, such a menace to his associates that he must forever be kept from mingling with healthy persons, why not, for the benefit of general mankind, chloroform him at once and save all this trouble, expense, and prolonged heartache? Is not euthanasia the proper principle upon which to proceed in the management of these cases? If not, why not?"

---

FLY SONG.

Ten little flies
All in a line;
One got a swat!
Then there were nine.
Nine little flies
Grimly sedate,
Licking their chops—
Swat! There were eight.
Eight little flies
Raising some more—
Swat! Swat! Swat! Swat!
Then there were four.
Four little flies
Colored green-blue;
Swat! (Ain't it easy!)
Then there were two.
Two little flies
Dodged the civilian—
Early next day
There were a million!

—*Buffalo News.*

[That swat's the matter.—ED.]

